# Screening for depressed mood in an adolescent psychiatric context by brief self-assessment scales – testing psychometric validity of WHO-5 and BDI-6 indices by latent trait analyses

**DOI:** 10.1186/1477-7525-10-149

**Published:** 2012-12-11

**Authors:** Eva Henje Blom, Per Bech, Göran Högberg, Jan Olov Larsson, Eva Serlachius

**Affiliations:** 1 Department of Clinical Neuroscience, Karolinska Institutet, Retzius väg 8, A2:3, Stockholm, 17177, Sweden; 2 Psychiatric Research Unit Mental Health Centre North Zealand, Dyrehavevej 48, DK 3400, Hillerød, Denmark; 3 Department of Women’s and Children’s Health, Building H2:00 Karolinska University Hospital, 171 76, Stockholm, Sweden; 4 Department of Women’s and Children’s Health, Karolinska Institutet, Retzius väg 8, A2:3, Stockholm, 17177, Sweden

**Keywords:** Well-being, Adolescent major depressive disorder, Rasch analyses, Mokken analyses, Brief self-assessment scales, BDI-6, WHO-5

## Abstract

**Background:**

Major depressive disorder is prevalent in the adolescent psychiatric clinical setting and often comorbid with other primary psychiatric diagnoses such as ADHD or social anxiety disorder. Systematic manual-based diagnostic procedures are recommended to identify such comorbidity but they are time-consuming and often not fully implemented in clinical practice. Screening for depressive symptoms in the child psychiatric context using brief, user-friendly and easily managed self-assessment scales may be of clinical value and utility. The aim of the study is to test the psychometric validity of two such scales, which may be used in a two-step screening procedure, the WHO-Five Well-being Index (WHO-5) and the six-item version of Beck’s Depression Inventory (BDI-6).

**Method:**

66 adolescent psychiatric patients with a clinical diagnosis of major depressive disorder (MDD), 60 girls and 6 boys, aged 14–18 years, mean age 16.8 years, completed the WHO-5 scale as well as the BDI-6. Statistical validity was tested by Mokken and Rasch analyses.

**Results:**

The correlation between WHO-5 and BDI-6 was −0.49 (p=0.0001). Mokken analyses showed a coefficient of homogeneity for the WHO-5 of 0.52 and for the BDI-6 of 0.46. Rasch analysis also accepted unidimensionality when testing males versus females (p > 0.05).

**Conclusions:**

The WHO-5 is psychometrically valid in an adolescent psychiatric context including both genders to assess the wellness dimension and applicable as a first step in screening for MDD. The BDI-6 may be recommended as a second step in the screening procedure, since it is statistically valid and has the ability to unidimensionally capture the severity of depressed mood.

## Introduction

Major depressive disorder (MDD) is the most prevalent psychiatric disorder in teenagers across cultures [1]. In teenagers MDD is highly comorbid with other psychiatric disorders such as generalised anxiety disorder, social anxiety disorder and ADHD and may have a negative impact on the prognosis and treatment outcome of the comorbid disorder if not identified and treated [1]. Systematic manual-based diagnostic procedures have been shown to help identify comorbid disorders in child and adolescent psychiatry [2] but are often time-consuming and not fully implemented in clinical practice. We suggest that screening for depressive symptoms using brief, user-friendly self-assessment scales in the child psychiatric context may be of additive clinical value and utility. A salutogenic scale has an obvious advantage in avoiding repetitive questions concerning negative affect and negative life experiences that may be discouraging for the patient and is therefore suggested as a first step when screening for depressive symptoms. We have focused on the WHO-5 as the initial screening instrument for the measurement of subjective well-being [3], see Appendix 1. Recently, Hall et al. examined the clinical validity of widely used well-being scales and identified the five-item WHO-5 scale as having the highest content validity when compared to scales with a much larger number of items such as the 22-item Psychological General Well-Being Index, the 36-item Medical Outcome Short Form (SF-36) or the 100-item World Health Organisation Quality of Life Scale (WHOQoL) [4]. In the follow-up discussion with mental health experts, Hall et al. state that it is often difficult to avoid the use of items reflecting either symptoms of illness or side-effects of medication such as sleep problems or concentration problems [4]. As a second step in the screening procedure, when the WHO-5 shows a score below a certain cut-off, an inventory specifically measuring the pure depressive symptom severity should be used. For this purpose we have focused on the short, six-item version (BDI-6) of the Beck Depression Inventory [5], see Appendix 2 in which six core items have been selected from the original version of Beck’s Depression Inventory to capture MDD unidimensionally in a brief format [6], thus avoiding unintentionally screening for symptoms which might be side-effects of medication. The aim of this study is to investigate the psychometric properties of the brief self-rating scales WHO-5 and BDI-6 in a clinical context of teenagers with MDD with or without other comorbid psychiatric diagnosis by item Rasch and Mokken analyses. A two-step screening approach is based on the fact that the positively phrased items (WHO-5) measure when “the wind begins to get taken out of the sails”, in other words an ultra-short screening before the syndrome of depression is in operation. The negatively phrased items (BDI-6) measure the cognitive theory of depression.

## Method

### Self-assessment scales

The WHO-Five Well-being Index (WHO-5) was derived from a larger rating scale developed for a WHO project on quality of life in patients suffering from diabetes [7]. During the first psychometric evaluation 10 of the original 28 items were selected due to the homogeneity they had shown across the various European countries participating in this study [7]. Because positive psychological well-being has to include positively worded items only, these 10 items were then reduced to five items (WHO-5) which still covered positive mood, vitality and general interest. The five items are: (a) being in good spirits, (b) feeling relaxed (c) having energy (d) waking up fresh and rested, (e) being interested in things. Each of the five items is rated on a 6-point Likert scale from 0 (= not present) to 5 (= constantly present). The theoretical raw score ranges from 0 to 25. Thus, higher scores mean better well-being. The raw score is obtained by adding the figures in the boxes. A score below 13 indicates poor well-being and is an indication for testing for depression, as is the case if the patient has answered 0 to 1 on any of the five items [3]. In order to monitor possible changes in well-being, the percentage score is used. The percentage value is calculated by multiplying the score by 4 and thus obtaining a scale from 0 (worst imaginable well-being) to 100 (best imaginable well-being). Conventionally > 50 is interpreted as indicating no depression, 30 – 50 mild depression and < 30 moderate depression. A 10% difference indicates a significant change [8]. Appendix 1 contains the full version of the items. The six items of the BDI-6 index were derived from the original version of Beck’s Depression Inventory BDI-21 in a clinical validation study [6], using experienced psychiatrists as index of validity, to capture major depressive disorder (MDD) in a brief format. In this process, 12 items were shown to follow the global severity index of the experienced psychiatrists. However, many of these 12 items had local dependency. Hence, the BDI items 1 (sadness), 2 (pessimism) and 4 (lack of satisfaction) showed a very high local dependency. Moreover, item 5 (guilt), item 3 (sense of failure) and item 6 (sense of punishment) also had a very high local dependency. On the other hand, the selected items in the BDI-6 have a high clinical correspondence to MDD, without being related to each other. The BDI-6 has not yet been tested or validated in a child psychiatric population. The six items are: (1) feelings of sadness, (5) feelings of guilt, (11) being irritable, (13) having decision problems, (15) ability to work and (17) feeling tired. When this study was designed, the BDI-II had not yet been validated for the Swedish version, while BDI-A1 was in use. The six BDI-6 items were retroactively extracted from the full 21-item BDI-A1 version. The BDI-6, as used in this study, thus contains six items on a 4-point scale yielding a total score by summation of the individual items of 0–18 p (0 p means no depression and 18 p maximum depression score). The conventional cut-off scores for BDI-6 are < 6 no depression, 6–7 mild depression, 7 moderate depression. Appendix 2 contains the full version of the items.

### Data collection procedure

The data collection was part of a larger study which has been described in detail elsewhere [9]. In this study 63 adolescents, 57 girls and 6 boys, aged 14–18 years (mean age 16.8 years) who were psychiatric patients with a clinical diagnosis of major depressive disorder completed the WHO-5 scale as well as the BDI A-1. The clinical diagnosis of MDD was validated by DAWBA, but since this study aimed at validating the WHO-5 and BDI-6 as a screening procedure and was not primarily aimed at studying a group of depressed teenagers all patients were included, both the ones in whom DAWBA validated the diagnosis of MDD and the ones who had a clinical diagnosis of MDD, which was not validated by DAWBA. Patients with severe autism or psychotic symptoms were not included in the study. The subjects had on-going treatment contact (median duration 11 months) at one of 13 outpatient psychiatric clinics for children and adolescents situated in the centre of Stockholm, its suburbs and in smaller towns nearby. The subjects completed the self- assessment forms at the open psychiatric units. An endeavour was made to collect all data on one occasion, but in some cases new appointments had to be made to complete all stages of the procedure. This involved testing one subject at a time under the supervision of one or two assistants. A randomly sorted pack of forms was handed to each subject, who was then free to choose in which order to complete them.

### Psychometric analyses

Both Mokken and Rasch methods are based on item response theory, thus assuming: (a) unidimensionality, meaning that for the items that form a scale there is assumed to be a dominant single latent trait that determines how the questions are answered, for WHO-5 perceived wellness and for BDI-6 depressed mood, (b) local stochastic independence of the items in a scale meaning that a specific individual’s response to items in a scale is dependent on the individual’s level of the trait being measured so that the score of one item is not explained by the score of another item. Local stochastic independence (conditional independence) should be distinguished from the problem of the clinical local independence of items. Statistically, local independence is concerned with how different item scores are stochastically related through independence for any fixed level of depression. Any specific item response is consequently determined solely by the location on the dimension [10] and the score level for the specific patient. However, clinically we are dealing with local independence as a measure (correlation) of the extent to which the score on one item can automatically predict the score on another item. The full BDI-21 contains, for example, many such logically correlating items, as discussed by Greenberg [11], and finally (c) monotonicity, referring to the probability of the score of the item increasing as the level of the latent trait increases [12,13]. Invariant item ordering is one of the key features and a basic principle underpinning the item response theory models. In these models it is stipulated that items with lower prevalence have to be preceded by items with higher prevalence for all patients under consideration [14]. The Rasch model provides a method for nonlinear transformation of ordinal raw scores to interval measures and allows the raw scores from different items representing different severity to be summated [15]. Estimated by means of the single-parameter Rasch model, the locations (item parameters) of the theoretical range of the latent dimension of depression at which the prevalence of items can be placed are illustrated in the tables. We used the RUMM 2030 program (Andrich D, Sheridan BE, Luo G. RUMM2030. version 5.1. Perth, WA: RUMM Laboratory Pty Ltd; 2010) to estimate the item locations.

Mokken analysis is considered a useful way of investigating the behaviour of items in scales in relation to varying levels of the latent trait, and the coefficient of homogeneity is considered to indicate unidimensionality if > 0.40. The Mokken analysis has been frequently applied to psychological self-assessment questionnaires [13]. In accordance with the Mokken model, the listing of the individual items is determined by the mean item scores. In the Mokken scale analyses we used a program for polytomous items created by Molenaar et al. [16]. Both the parametric Rasch item response model and the non-parametric Mokken model are only relevant to use if the scales under examination (WHO-5 and BDI-6) have acceptable clinical validity. The psychometric validation by Rasch and Mokken is a validation of the measurement aspect answering the question whether the total score is a sufficient statistic.

The rationale of using both Rasch and Mokken analyses rather than one or the other is that the Mokken model shows the coefficient of homogeneity which is easy to understand, but with the Rasch model we can check the influence of external factors, e.g. gender.

## Results

**Figure 1:**
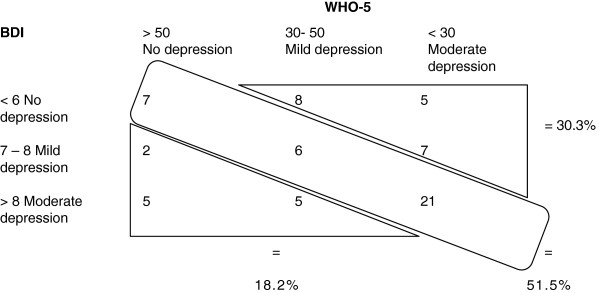
ROC analysis showing the results of WHO-5 and BDI-6 of the total subjects (N=66) with clinical diagnosis of major depressive disorder.

**Figure 2:**
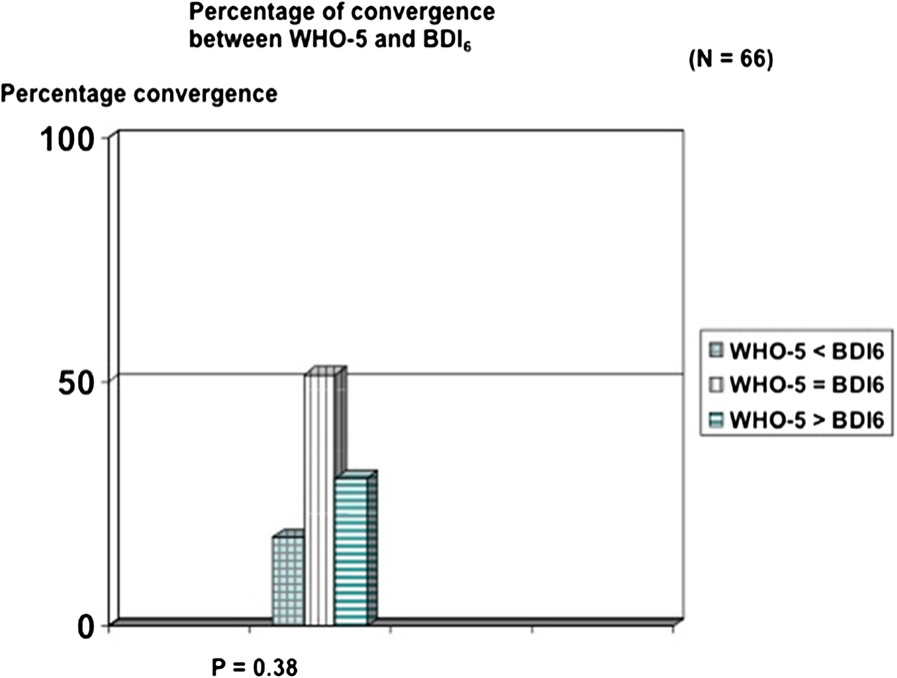
Showing the percentage of convergence between WHO-5 and BDI-6.

Of the 66 patients, 40 obtained a diagnosis of MDD when validated by DAWBA. Using this diagnosis as index, we found for WHO-5 with the conventional cut-off score of < 50 a sensitivity of 0.80 and a specificity of 0.29. Using the conventional cut-off score for BDI-6 of < 7 for moderate depression, we found a sensitivity of 0.50, but a specificity of 0.83. An ROC analysis confirmed the strategy of the two-step screening procedure for depression by WHO-5 and BDI-6 (Figure 1). WHO-5 and BDI-6 were not without convergence (P = 0.38), corresponding to a Spearman coefficient of −0.49 (P < 0.01). However, in 30.3% of cases, WHO-5 indicates that a BDI-6 depression is in operation before the BDI-6 cut-off score is reached (Figure 2). The total score of the five items in the WHO-5 is a sufficient statistic with a coefficient of homogeneity of 0.52 (Table 1). Table 1 shows the means (rankings) for the Mokken analysis of the WHO-5 scale. The three “psychological” items (item 1, 2, and 5) have the highest rankings, indicating that they are present in low to moderate degrees of well-being. The two “somatic” items (4 and 3) have the lowest rankings indicating that they are only present when high degree of well-being is perceived. Table 2 shows the Rasch analysis for the WHO-5 with the three “psychological” items having the highest locations (up to minus 1.13) and the two “somatic” items with the lowest rankings. The Rasch analysis also accepted unidimensionality when testing males versus females (p > 0.05). The coefficient of homogeneity for the BDI-6 was 0.46. Table 3 shows the means (rankings) for the Mokken analysis of the BDI-6. The two “somatic items” (17 and 15) showed the highest rankings, indicating that they are present in low to moderate degree of depressed mood and the “psychological items” (13, 1, 5, 11) showed the lowest, indicating that they are only present in a more severely depressed state. Table 4 shows the Rasch analysis with the items of “psychological asthenia” or “apathy” having the highest prevalence (items 17 and 15). The Rasch analysis also accepted unidimensionality when testing males versus females (p > 0.05). The correlation (Spearman) between BDI-6 and WHO-5 was −0.49 (p=0.0001).

**Table 1:** Mokken analysis of WHO-5 showing the means and items (rankings)

	**Item 1**	**Item 2**	**Item 5**	**Item 4**	**Item 3**
	Good spirits	Relaxed	Interested in things	Fresh and rested	Energy
	2.22 (1)	1.84 (2)	1.70 (3)	1.12 (5)	1.52 (4)
Coefficients of homogeneity*	0.11	0.52	0.40	0.53	0.49

*The corresponding coefficient of homogeneity for the total score of WHO-5 (= 0.52).

**Table 2:** Rasch analysis of WHO-5 showing the locations (ranks)

**Item 1**	**Item 2**	**Item 5**	**Item 4**	**Item 3**
Good spirits	Relaxed	Interested in things	Fresh and rested	Energy
−1-13 (1)	- 0.26 (2)	- 0.22 (3)	0.31 (4)	1.30 (5)

**Table 3:** Mokken analysis of the BDI-6 showing the means and items (rankings)

	**Item 17**	**Item 15**	**Item 13**	**Item 1**	**Item 5**	**Item 11**
	Tiredness	Work	Decision problems	Sadness	Guilt	Irritability
	1.54 (1)	1.48	1.21 (3)	1.14 (4)	1.12 (5)	1.00 (6)
Coefficients of homogeneity*	0.49	0.49	0.48	0.40	0.44	0.47

*The corresponding coefficient of homogeneity for the total score of BDI-6 (=0.46).

**Table 4:** Rasch analysis of the BDI-6 showing the locations (ranks)

**Item 17**	**Item 15**	**Item 13**	**Item 1**	**Item 5**	**Item 11**
Tiredness	Work	Decision problems	Sadness	Guilt	Irritability
- 0.26 (1)	- 0.23 (2)	0.03 (3)	0.13 (4)	0.16 (5)	0.17 (6)

## Discussion

This study shows that the brief self-assessment scales WHO-5 and BDI-6 have a satisfactory statistical validity in a sample of adolescents of both genders with a clinical diagnosis of major depressive disorder (MDD) with or without comorbidity of other psychiatric disorders. The Mokken analysis of the WHO-5 scale shows a coefficient of homogeneity of 0.52 indicating unidimensionality of the latent trait of wellness; the analysis of BDI-6 showed a coefficient of homogeneity of 0.46, indicating that the total score is a sufficient measure of the pure dimension of depressed mood. These findings confirm the statistical validity of using the WHO-5 as a first step in a screening procedure for depressed mood among adolescent psychiatric patients. The total score WHO-5 and BDI-6 showed an inverse relationship of -0.49 (p<0.0001) indicating that the two scales partially overlap, but that the wellness dimension captures other aspects of well-being than the absence of depressed mood. Therefore, BDI-6 is needed as a brief scale that adequately measures the dimension of depressed mood as a second step when the WHO-5 is below a certain cut-off. However, we do not suggest this procedure as an alternative to a proper manual-based diagnostic interview, which would capture other comorbid psychiatric disorders in addition to MDD, but rather as a convenient and user-friendly complementary way to screen specifically for depressed mood in situations when the diagnostic interview is too time-consuming, impractical or inappropriate to use. Such situations may be when the patient fails or refuses to cooperation or when it is too much an effort for the patient to manage a diagnostic interview. In such cases it is an advantage to have brief self-assessment scales with a salutogenic focus as a first screening tool. The brief scales may of course be used for purposes other than for screening. The WHO-5 can be used across diagnoses to assess well-being as an aspect of quality of life, either on specific occasions such as intake and after treatment or in session-by-session assessment, i.e. treatment monitoring [17]. The WHO-5 scale has been chosen in this study because it specifically aims to measure positive well-being, in contrast to other short scales for the measurement of quality of life focusing on other aspects of health status including morbidity, self-care, pain etc. The BDI-6 as a unidimensional measure of depressive symptoms is valid for assessment of treatment outcome in MDD. Interestingly, the BDI-6 items of sadness, guilt and irritability were reported more frequently as the severity of depressive symptoms increased, whereas tiredness and decreased ability to work had the highest ranking, indicating that these symptoms are present even when the depressed mood is mild to moderate. The same tendency was found from a wellness perspective with the WHO-5. This indicates that depressive symptoms present themselves in a certain sequence so that in a mild form the dominant symptoms are tiredness and subjectively reduced ability to work and when the severity increases feelings of sadness, guilt and irritability occur. This data supports the theory of Hamilton that mental illnesses, especially depression, manifest themselves, so that the patients are the first to experience a disturbance of their subjective well-being. Subsequently, family relationships and later their wider social adjustment are impaired, often with increasingly negative impact on their global functioning [18].

The study population was limited to patients with mild to moderate depression and did not include any hospitalised psychiatric patients, which limits the generalisability of the results to the full spectrum of adolescent psychiatric patients. The self-assessment forms were distributed in a random order to prevent systematic errors, but there could still be an effect of having to complete several different questionnaires. The BDI was presented to the subjects as the full 21-item BDI-1A version and the items of the BDI-6 were retroactively selected. Consequently, we cannot be certain that the scoring of the six items would have been identical if the BDI-6 had been presented instead of the full-items version. A few subjects occasionally mark some items of the BDI-21 between the specified numbers of 0–4, so that the scores had to be interpreted as half numbers (0,5; 1,5; 2,5). We therefore used a 0–6 Likert scale in the analyses, but in the Mokken mean scores we went back to a 0–3 Likert scale by dividing the scores by 2 for comparison with the conventional BDI studies.

## Conclusions

We conclude that the WHO-5 is psychometrically sound and applicable in an adolescent psychiatric context of clinically depressed teenagers to assess the dimension of wellness. The WHO-5 scale has the advantage of a salutogenic approach and could be used as an initial screening instrument for depressed mood in a clinical context. The BDI-6 may be recommended as a second step in the screening procedure since it is statistically valid and adequately captures the severity of MDD.

## Appendix

### Appendix 1 **WHO (Five) Well-being Index (1998 version)**

Please indicate for each of the five statements which is closest to how you have been feeling over the last two weeks (Table 5). 

**Table 5:** WHO (Five) Well-being Index (1998 version)

	**Over the last two weeks**	**All of the time**	**Most of the time**	**More than half of the time**	**Less than half of the time**	**Some of the time**	**At no time**
1	I have felt cheerful and in good spirits	5	4	3	2	1	0
2	I have felt calm and relaxed	5	4	3	2	1	0
3	I have felt active and vigorous	5	4	3	2	1	0
4	I woke up feeling fresh and rested	5	4	3	2	1	0
5	My daily life has been filled with things that interest me	5	4	3	2	1	0

### Appendix 2 **The BDI-6subscale for depression**

1.

 A. I do not feel sad (0 p)

B. I feel sad and depressed (1 p)

C. I feel constantly sad and depressed and feel unable to get   out of it (2 p)

D. I feel so blue and unhappy that I cannot bear it (3 p)

5.

 A. I don’t feel particularly guilty (0 p)

B. I feel bad or unworthy a good part of the time (1 p)

C. I feel quite guilty (2 p)

D. I feel constantly as though I am guilty and worthless (3 p)

11.

 A. I am no more irritable now than I ever was (0 p)

B. I get annoyed or irritable more easily than I used to (1 p)

C. I feel irritated all the time (2 p)

D. I get irritated about things that did not use to irritate me   at all (3 p)

13.

 A. I make decisions about as well as ever (0 p)

B. I try to put off making decisions (1 p)

C. I have great difficulty in making decisions (2 p)

D. I cannot make any decisions at all anymore (3 p)

15.

 A. I can work about as well as before (0 p)

B. It takes extra effort to get started at doing something (1 p)

C. I have to push myself very hard to do anything (2 p)

D. I can’t do any work at all (3 p)

17.

 A. I don’t get more tired than usual (0 p)

B. I get tired more easily than I used to (1 p)

C. I get tired from doing anything (2 p)

D. I get too tired to do anything (3 p)

## Competing interests

All the authors declare that they have no competing interests.

## Authors’ contributions

EHB was the “project leader” for the study, responsible for the data collection, and wrote the first draft of the manuscript. PB contributed with statistical analyses and references to the WHO-5 and BDI-6. GH contributed to the original outlines of the project literature search. JOL was responsible for psychometric references and for methodological issues. ES contributed to the recruitment and diagnostic issues of the clinical sample and was responsible for the overall design of the study. All the authors contributed to and have approved the final manuscript.
